# Perception and argumentation in the LK-99 superconductivity controversy: a sentiment and argument mining analysis

**DOI:** 10.1038/s41598-025-98554-3

**Published:** 2025-04-17

**Authors:** Eunhye Kim, Wei Luo, Hunkoog Jho

**Affiliations:** 1 Institute of Knowledge Integration & Design, Dankook University, Yongin, Korea; 2 School of Information Technology, Deakin University, Melbourne, Australia; 3 Department of Science Education, Dankook University, 152, Jukjeon-ro, Suji-gu, Yongin-si, Gyeonggi-do Korea

**Keywords:** Sentiment analysis, Argument mining, Public Understanding of science, Superconductivity, LK-99, Social media analysis, Superconducting devices, Energy and society, Sustainability, Risk factors

## Abstract

The announcement of LK-99 as a potential room-temperature, ambient-pressure superconductor sparked widespread debate across both traditional news outlets and social media platforms. This study investigates public perceptions and argumentation patterns surrounding LK-99 by applying sentiment analysis and computational argument mining to a diverse dataset. We analyzed 797 YouTube videos, 71,096 comments, and 1,329 news articles collected between 2023 and 2024. Our results reveal distinct sentiment trajectories: while news articles and YouTube posts exhibit fluctuating yet predominantly positive tones, user comments consistently maintain a more negative sentiment. Discourse analysis shows that structured argumentation—especially reasoning based on expert opinions, observable signs, and anticipated consequences—is prevalent in professionally curated content, whereas a significant proportion of user comments lack identifiable argumentation schemes. Moreover, channel-level analysis indicates that non-expert channels, despite their limited specialization in science, attract higher audience engagement than traditional science channels. These findings highlight the complexities of digital science communication and underscore the need for adaptive strategies that bridge the gap between expert evidence and public discourse. Our study provides practical recommendations to enhance public understanding of scientific advancements in digital spaces.

##  Introduction

As science increasingly influences and intersects with various societal, environmental, health, political, and economic problems, various scientific issues become the focus of substantial public discussions. Representative examples include climate change, genetically modified foods, and vaccine safety. Publics tend to engage more with scientific issues that directly impact their daily lives, and this interest leads them to seek more information and contribute their views, experiences, and thoughts to broader discussions^1^. Through this process, people exchange knowledge, share perspectives, and collectively construct social interpretations of these issues^2^. Therefore, public discourse around these issues often plays a critical role in shaping plans and decisions regarding new policies^3,4^.

Social media can contribute to public engagement in science by enabling information sharing, diverse expressions, and dynamic discussions, which can make scientific issues more accessible to broader audiences^5,6^. However, it is important to recognize that social media can also make public discussions more complex by spreading misinformation, reinforcing echo chambers, and contributing to polarization^2,7–10^. For instance, recent studies have demonstrated that false news tends to spread faster and wider than true news and that the COVID-19 infodemic has further amplified public polarization^11,12^.

The discrepancies between public and expert reactions can sometimes lead to significant societal scandals, as seen in the case of Theranos, a healthcare technology startup. The company’s founder, Elizabeth Holmes, claimed to have developed a revolutionary blood-testing device capable of diagnosing multiple diseases from a single drop of blood at home. This promise gained widespread attention—particularly through social media—with publics reacting enthusiastically to the vision. As Liang et al.^13^ note, new media environments can effectively build buzz around scientific ideas, which may have contributed to the rapid spread of Holmes’s claims. However, medical scientists raised concerns early on, questioning the feasibility of the technology due to significant technical limitations. Despite these warnings, the media hype overshadowed expert skepticism, and substantial investments were funneled into the company over several years. Ultimately, experts in laboratory medicine and investigative journalists exposed Theranos’s fraudulent practices, revealing that the promised technology was never viable^14,15^.

In South Korea, there was significant public debates surrounding the discovery of LK-99, which drew comparisons to the Theranos scandal. In July 2023, a South Korean research team claimed that LK-99 was a superconductor that worked at room temperature and ambient pressure—a breakthrough that, if validated, could have profound societal and practical implications. For instance, such a superconductor could revolutionize quantum computing by drastically reducing energy losses, enhance magnetic levitation systems for transportation, and improve the efficiency of medical imaging technologies. The name ‘LK-99’ originates from the initials of the researchers who led its development and the year it was first created. ‘LK’ represents the surnames of Sukbae Lee and Jihoon Kim, while ‘99’ signifies the year 1999, when the material was initially developed. Although LK-99 was developed in 1999, the recent announcement in 2023 quickly went viral across social media, sparking both excitement and skepticism. Many members of the South Korean publics, particularly those enthusiastic about its potential social and economic impacts, anticipated major technological advancements, which also led to fluctuations in the South Korean stock market^16^. On the other hand, material scientists and superconductivity researchers expressed doubts, citing concerns about the material’s structure and identity^17,18^. Experts and non-experts participated in discussions on social media, sharing experimental reports, articles, and videos. These debates continued for over two years, even though a consensus eventually emerged that LK-99 does not exhibit superconducting properties.

Traditionally, models such as the two-step flow or deficit model suggested that information flowed in a pipeline from experts to publics. However, with the advent of digital media and social networking platforms, communication dynamics have evolved into a networked public sphere where active participation and diverse perspectives shape public opinions. In this environment, phenomena such as filter bubble and echo chamber effects have emerged to explain how algorithm-driven content curation and online community dynamics can reinforce selective exposure and cognitive biases^11,19^.

In this study, *sentiment* refers to the emotional tone expressed in textual content—categorized as positive, neutral, or negative—while *argumentation schemes* are defined as structured patterns of reasoning following Douglas Walton’s framework. Previous research in science has shown that digital media can both enhance public engagement and contribute to information polarization^2,5^. These insights, along with studies on public sentiment formation in digital environments^20,21^, underscore the need to revisit traditional models of science communication in light of emerging networked communication dynamics.

Given the controversies surrounding cutting-edge scientific issues such as LK-99, it is crucial to examine how public perceptions are shaped in the digital age and how their knowledge and opinions were aligned with or different from those of experts. This study aims to bridge that gap by applying sentiment analysis and argument mining to media and social media data, thereby providing a nuanced understanding of science communication dynamics and offering insights for more effective public engagement strategies.

This research zooms in on public discussions about LK-99, especially focusing on how people have perceived and responded to this scientific topic on YouTube. To explore people’s subjective and evolving perceptions and reactions regarding scientific issues, previous studies have analyzed how emotions shift between positive, neutral, and negative sentiments^22–24^ or what reasoning strategies people employ to construct and justify arguments in their social media posts^1^. This research also investigates people’s perceptions of and reactions to LK-99 by analyzing their sentiments and argumentation schemes reflected in their posts. This research also considered the different types of participants in public discussions, including journalists who write and post news articles, YouTube creators who produce videos, and commenters who engage with the videos by commenting rather than simply liking or disliking^20,21^.

We selected news articles because they provide a formal, professionally curated narrative of the LK-99 controversy, while YouTube content captures a heterogeneous mix of voices—including ordinary citizens, influencers, science communicators, and experts—that reflect the broader public discourse. By comparing these two channels, our study aims to highlight potential discrepancies between structured media reporting and the diverse, informal opinions circulating on social media. While we acknowledge that YouTube content is authored by a mix of individuals (ranging from journalists to ordinary citizens), this diversity is central to our analysis of public sentiment and argumentation. It allows us to capture the multifaceted nature of science communication in digital spaces. Although future work may explore filtering YouTube content by author background to isolate purely public communication, our current approach intentionally preserves the diverse spectrum of opinions to reflect the real-world complexity of online discourse. In this light, this study seeks to address the following questions:

### Q1

How do public sentiments toward emerging scientific claims develop and shift over time across different media ecosystems, and what key events or factors drive these sentiment trajectories?

### Q2

What argumentation schemes were used in these three types of social media sources?

## Method

The present study aims to deepen our understanding of public perceptions surrounding the LK-99 controversy by examining how sentiments are expressed and evolve, as well as by identifying the argumentation schemes employed across different media channels. To achieve this objective, we collected and analyzed content from news articles, YouTube posts, and YouTube comments over the period from July 23, 2023, to August 23, 2024. This time frame was chosen because it captures the peak period of public discourse on LK-99. Our preliminary analyses indicated that, as time progressed, the frequency of relevant content decreased dramatically—falling to fewer than 10 items after August 2024—thus justifying the upper limit of our data collection period.

**Fig. 1:**
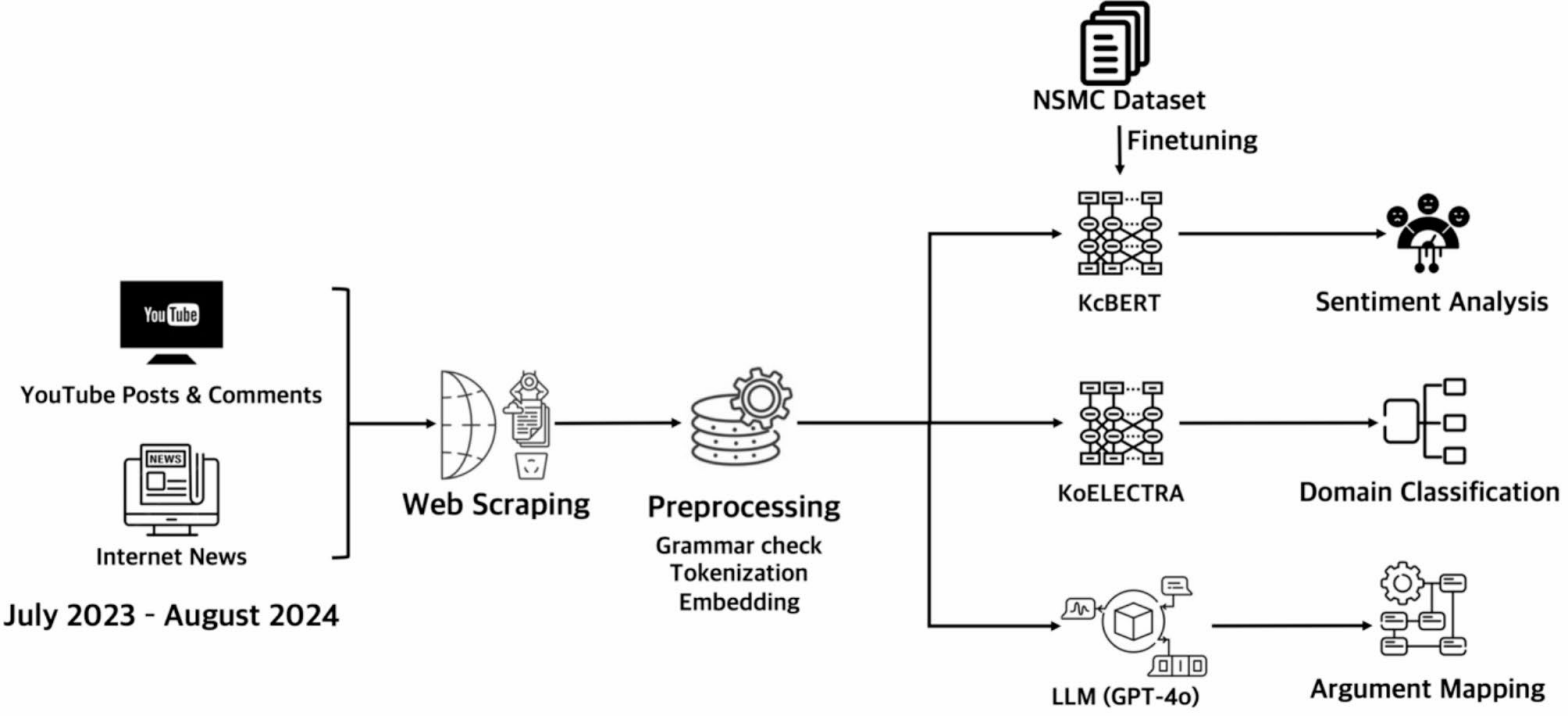
Outlines the research workflow.

Figure 1 Research Workflow for Sentiment Analysis, Domain Classification, and Argument Mining on YouTube and News Data.

Data were collected using the YouTube Data API, focusing on posts containing ‘LK-99’ and ‘room-temperature superconductor.’ In the preliminary stage, we evaluated a range of keywords—including superconductivity, high-temperature superconductor, and superconductor—but ultimately selected these two terms to extract data most directly related to the LK-99 announcement. All data were in Korean. Related metadata and transcripts were also retrieved. YouTube comments related to LK-99 were collected using a Python-based web crawler, which retrieved details such as the comment user IDs, comment content, timestamps, and the number of likes^20,25^. While the YouTube API provides access to a large volume of publicly available posts, it may not capture every relevant post due to inherent limitations such as API restrictions and algorithmic filtering.

News articles dealing with LK-99 were retrieved from the Korea Press Foundation’s web portal. Established in 2010 under the ‘Promotion of Newspapers Act,’ this quasi-governmental organization comprises most domestic media outlets. It plays a crucial role in ensuring stable funding and quality reporting through government advertising, news gathering, and production support programs, and it provides comprehensive and reliable access to media statistics, research reports, and articles. This makes it unnecessary to individually access separate news agencies or portals for a complete view of domestic news. The Korea Press Foundation’s portal offers comprehensive coverage of domestic news articles. These factors were considered when interpreting the results of our study.

A total of 797 YouTube videos, 71,096 comments, and 1,329 news articles were included in the analysis. We applied specific inclusion criteria to ensure that only posts directly related to LK-99 were retained, while exclusion criteria were used to filter out irrelevant content. Data cleaning involved standardizing formats, correcting errors, and normalizing Korean text, ensuring high-quality inputs for subsequent analyses. The validity and reliability of our analyses were further supported by rigorous methods, such as 5-fold cross-validation for sentiment analysis (yielding an accuracy of 80.3%) and other accuracy metrics for domain classification and argument mining^26^.

An important criterion for understanding public perceptions about LK-99 is the overall polarity of opinions expressed about the issue—whether sentiments tend to be positive, negative, or neutral. Sentiment analysis, as a widely adopted method in social media research^27,28^, provides valuable insights into these evaluative responses and helps elucidate publics’ attitudes toward scientific controversies. Therefore, this study aimed to analyze the sentiment expressed in YouTube posts, comments, and news articles addressing LK-99 and to compare these sentiments across different media types. To examine public opinions toward LK-99, we conducted sentiment analysis by categorizing sentiments as positive, neutral, or negative. A BERT-based model, fine-tuned from KcBERT, was employed. Built upon the Transformer architecture as described by Vaswani and others^29^, this model leverages pre-trained language representations and self-attention mechanisms to capture subtle contextual nuances in text, such as idiomatic expressions and sentiment-bearing phrases. During fine-tuning, the model was adapted to our specific sentiment analysis task using the Naver Sentiment Movie Corpus (NSMC), which comprises 200,000 sentiment-labeled samples. Although we acknowledge that a three-tier classification may not capture all nuances of public opinion, it serves as a robust baseline for comparing sentiment across media types. Future research could build on this framework by incorporating more fine-grained sentiment and emotion analysis methods.

Based on established frameworks and empirical studies on news categorization—including the Reuters/RCV1 corpus^30^, the comprehensive review of categorization presented in the previous studies^31,32^—we derived six news categories for our study. By integrating insights from these sources and fine-tuning our approach to better reflect the thematic diversity observed in our dataset, we established the following six categories: science, technology, economy, politics, society, and ethics. These categories were chosen as they best capture the range of topics present in the analyzed news content. A KoELECTRA-based model was fine-tuned on a custom dataset created from our collected data, achieving an accuracy of 85.1%^34^. Domain classification is widely used to understand the thematic focus of large datasets and to track shifts in discourse over time^6^. We note that while this classification offers a useful baseline for categorizing content, it may not capture every nuance of individual engagement.

To understand the basis for positive or negative judgments about LK-99 by the media and publics, argumentation elements were extracted and analyzed. To examine the reasoning structures underlying sentiments expressed about LK-99, we employed argument mining based on Douglas Walton’s argument schemes^34^. Walton’s framework comprehensively categorizes common patterns of reasoning found in everyday argumentation. After reviewing this framework and considering several prior studies in computational argument mining that identified key argument schemes relevant to public discourse^6,34^, we selected ten schemes that were most significant for our analysis: argument from expert opinion, analogy, cause to effect, sign, example, popular opinion, consequence, commitment, ignorance, and value. The automated classification was performed using the GPT-4 API, following established methodologies in computational argument mining that emphasize the use of predefined argument schemes, robust natural language processing techniques, and iterative validation to ensure accuracy and relevance^2,35,36^.

For YouTube videos, while multiple arguments may be present, our analysis focused on identifying the dominant argumentation scheme within each video. This approach allows us to capture the primary mode of reasoning that characterizes the video’s overall message. In contrast, for YouTube comments—which tend to be shorter and less complex—we analyzed each comment individually, often yielding a single identifiable argumentation scheme per comment. This method enables a direct comparison of argumentation patterns across media types, despite the inherent differences in content complexity.

To investigate the relationship between sentiment polarity and the use of specific argumentation schemes, we conducted correlation analyses using Pearson’s correlation. These analyses were performed using Python libraries with a significance level set at *p* < 0.05. The results of these tests helped us determine the extent to which shifts in sentiment were associated with variations in argumentation patterns.

To further examine how argumentation schemes vary based on the publication sections, we categorized news articles and YouTube Posts according to their content sections (e.g., scientific, economic, technological). We then analyzed the distribution of argumentation schemes within each category. This analysis was conducted using Pearson’s correlation to determine whether the use of specific argumentation schemes significantly differed across sections. A significance level of *p* < 0.05 was adopted for all statistical tests.

The results were visualized to highlight monthly trends in sentiment across YouTube Posts, comments, and news articles. We analyzed the correlation between sentiments in YouTube posts and their corresponding comments and compared argumentation styles by media type and domain. This integrated approach reveals nuanced patterns in public engagement with scientific controversies^4,24^.

## Results

### Trend of three different sources

**Fig. 2:**
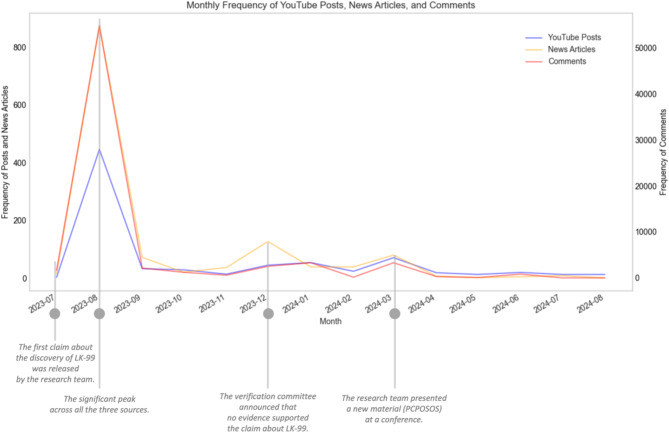
Monthly count of news articles, YouTube Posts, and YouTube comments.

Our analysis of frequency trends reveals that the number of posts surged in conjunction with key events but generally declined. Figure 2 illustrates the number of news articles (orange), YouTube Posts (blue), and comments (red) published online from 23 July 2023 to 23 August 2024. This dual-axis bar chart uses the left axis to represent the number of news articles and YouTube Posts, and the right axis shows the number of YouTube comments. When the first claim about the discovery of LK-99 was released in July 2023, there was a significant peak across all three categories: 874 news articles, 446 YouTube Posts, and 54,771 comments were published in August 2023. However, these numbers sharply declined after August and continued to drop until October 2023. Toward the end of 2023, there was a slight increase across all three categories, followed by another decline after the start of 2024. Although a modest rise was observed in March 2024, the overall posting frequency remained relatively low for all three groups through mid-2024. These temporal trends provide essential context for our analysis, as they help explain how fluctuations in discourse volume are associated with shifts in public sentiment and argumentation patterns over time, which is critical for understanding the evolution of public engagement with the LK-99 controversy.

The three groups’ posting frequencies commonly rebounded in December 2023 and March 2024, indicating that specific events related to LK-99 took place at these time points. On December 13, 2023, the Korean Society of Superconductivity and Cryogenics Verification Committee announced that no evidence supported the claim that LK-99 is a room-temperature, ambient-pressure superconductor. Since August 2023, the committee had been conducting verification procedures—obtaining samples of LK-99 from the Quantum Energy Research Centre and performing cross-measurements and replication studies based on methods outlined in the preprints. As a result, they concluded that LK-99 does not exhibit superconductivity at either room temperature or low temperatures, instead behaving as a non-superconductor with significant electrical resistance. Our content analysis of news articles, YouTube posts, and comments indicates that discussions focusing on these verification results became notably more frequent in December 2023. Later, On March 4, 2024, the Quantum Energy Research Centre research team presented a material called PCPOSOS at the American Physical Society. They claimed that this new material—created by adding sulfur to LK-99—exhibits room-temperature and ambient-pressure superconductivity. During the presentation, the research team detailed how the addition of sulfur altered LK-99, contrasting with the earlier findings of the Verification Committee. This announcement triggered another wave of discussions, as reflected by a surge in news articles, YouTube posts, and comments addressing the conference presentation and the new claim.

In addition to overall posting trends, our analysis of channel-level characteristics reveals notable differences between legacy and new media. A supplementary table (Table 1) summarizes key metrics—such as content frequency, average view counts, and likes—across channels, with legacy media (media = 1) generally exhibiting higher engagement than new media (media = 0). In our analysis, we considered only legacy media (i.e., the science channel or legacy media) as the expert group. These channel-specific differences may partly explain the variations observed in sentiment and argumentation patterns across our dataset. For instance, channels with higher engagement tend to feature more structured discourse and positive sentiment, which is reflected in our subsequent sentiment and argumentation analyses.

**Table 1:** Top 10 channels by content frequency and engagement metrics.

Rank	Channel Title	Primary Field	Number of Contents	Total View Count	Total Like Count	Total Comment Count
1	Notting King	Entertainment	1	9538.6 K	190,231	1,520
2	Romantic Park Sang-hwan	Lifestyle	1	6364.1 K	162,949	1,004
3	Sony Show Game Broadcasting	Gaming	1	5730.9 K	107,465	944
4	Judungi Broadcasting Shorts	Entertainment	1	3960.4 K	106,338	575
5	Weirdo TV	Comedy	1	3478.3 K	58,630	488
6	SBS News	Legacy News	32	6426.2 K	46,634	11,121
7	YTN	Legacy News	21	5429.1 K	34,390	7,259
8	Knowledge Pig Kkuing Kkuing	Information	1	2366.5 K	31,640	800
9	Jisikin Minani	Knowledge Sharing	4	2329.9 K	31,090	1,297
10	Comment Feast	Entertainment	1	1242.6 K	26,434	947
11	Info Boy	Information	3	1729.9 K	23,501	797
12	Owl’s Review	Media	3	1871.0 K	23,109	1,121
13	Thunderf00t	Science	1	346.6 K	20,583	1,251
14	Chimchak Man	Commentary/Gaming	1	2354.1 K	20,361	941
15	1-Minute Story Restaurant	Short Narrative	1	1015.5 K	17,177	279
16	Kim Junpyo	Entertainment	1	1422.3 K	14,761	221
17	Hot PD	Media	21	1210.8 K	14,426	2,535
18	Geekble	Science	1	1157.2 K	13,901	843
19	Unrealscience	Science	2	772.0 K	13,691	1,624
20	Jeon In-gu Economic Research Institute	Economy/Stock	2	449.8 K	12,961	1,188

To further explore how attention is distributed among different types of content creators, we examined the top 20 channels ranked by total likes and views for LK-99-related videos (Table 1). Among these channels, only three specialize in science, and two represent traditional legacy media, while the majority do not appear to have a direct focus on scientific reporting. In the analysis, we considered only these types of channels experts’ content. Although legacy media channels produced a considerable amount of LK-99 coverage, non-specialist channels collectively garnered higher view counts and likes, suggesting that producing a large volume of content or having recognized expertise does not necessarily guarantee high engagement. Notably, the top 20 channels accounted for only 12.58% of the total LK-99-related videos but attracted 75.95% of all views and 77.98% of all likes—indicating a strong concentration of audience attention. These findings raise questions about how effectively scientific information is communicated and received on social media platforms, particularly when a significant portion of engagement is driven by channels without a clear science focus.

### Sentiments

**Fig. 3:**
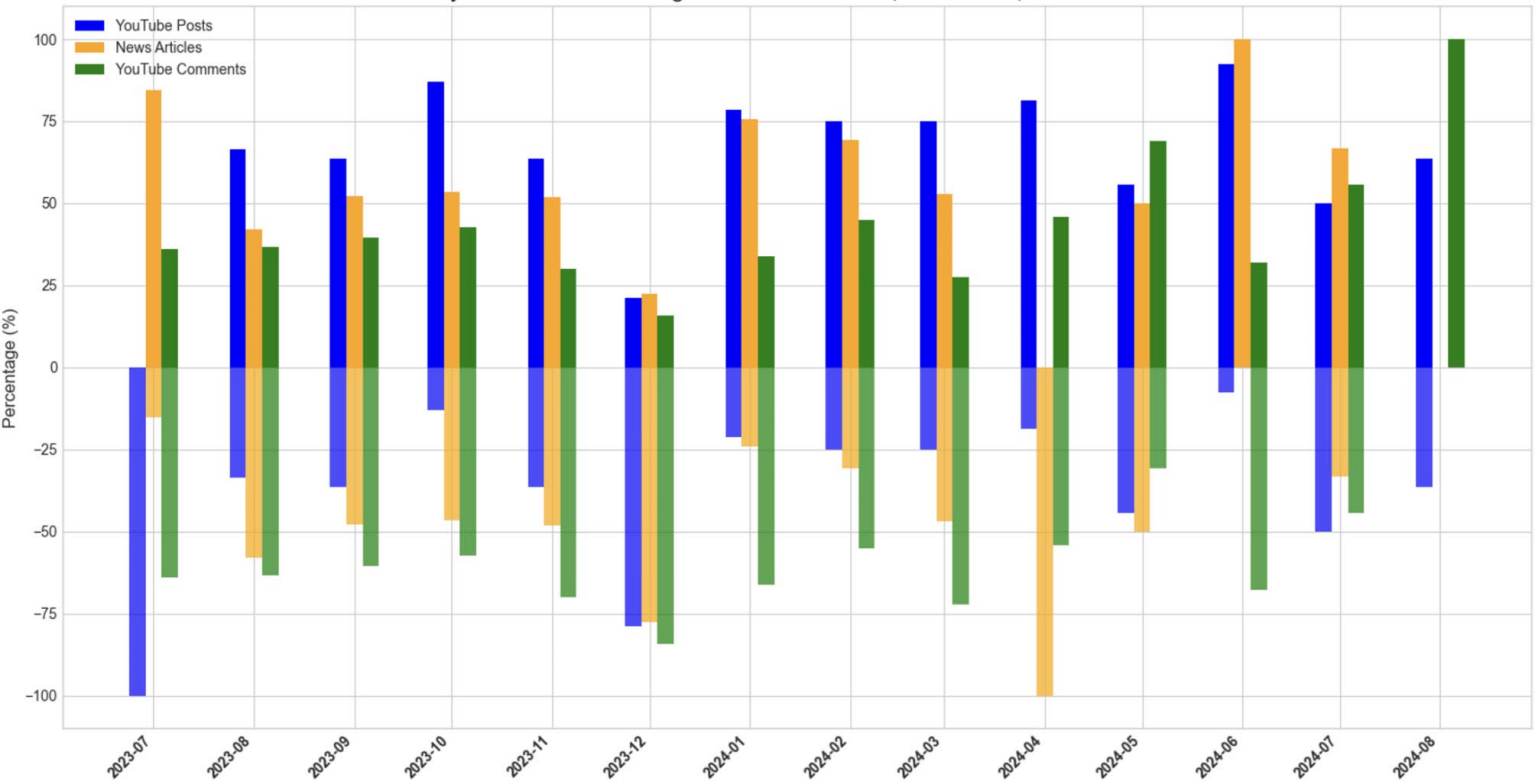
Sentiments revealed in news articles, YouTube Posts, and YouTube comments.

Our sentiment analysis of the three group posts shows that their sentiment orientations often exhibited similar trends during certain periods, but sometimes they displayed very different patterns. We categorized their posts into three types of sentiments: positive, negative, and neutral. Neutral sentiment posts did not include emotional expressions. Figure 3 displays how the posts were categorized into positive and negative sentiments in a dual-axis bar chart. The vertical axis represents the percentage of monthly positive(+ y) and negative(-y) responses about each type of source. This figure illustrates sentiment orientations and fluctuations across the three groups over the two years.

News articles exhibited slightly positive sentiments overall but showed significant volatility. Positive sentiment peaked in July 2023 following the release of LK-99 preprints but shifted to negative in August due to skepticism from academic communities and its impact on the stock market. Sentiments stabilized from September to November 2023 but turned negative again in December when the Verification Committee refuted LK-99’s superconductivity claims. A positive trend was observed in early 2024, coinciding with new material announcements from the research team, but declined again in April when academic skepticism resurfaced. Despite fluctuations, sentiments remained predominantly positive as articles highlighted the potential technological and economic impacts of Korean superconductor research.

YouTube Posts displayed a predominantly positive sentiment throughout the observed period, with fluctuations in the proportion of positive and negative sentiments each month. Initially, a high proportion of negative posts reflected skepticism from the research community regarding LK-99. However, positive sentiments gained prominence from September to November 2023 as creators focused on the potential scientific and technological advancements of room-temperature superconductors. In December 2023, the proportion of negative Posts spiked following the Verification Committee’s conclusion refuting LK-99’s superconductivity claims. Positive sentiment regained dominance in early 2024 as creators highlighted the announcement of new materials, but a slight decline was observed in April 2024 due to renewed skepticism. Despite these variations, positive sentiments consistently outweighed negative sentiments in most months.

YouTube comments predominantly exhibited a stable proportion of negative sentiments across most months, with fewer fluctuations compared to Posts or news articles. Negative comments were common early on, as users frequently pointed out the lack of verification for LK-99’s claims and skepticism from the research community. Positive sentiment proportions increased briefly in February 2024 following the announcement of PCPOSOS, a material claimed to improve LK-99 but declined again in subsequent months. Small positive spikes reappeared in May and August 2024, reflecting optimism about the broader potential of room-temperature superconductors. However, the overall sentiment remained predominantly negative throughout the period.

### Argumentation

Our analysis of argumentation schemes embedded in news articles, YouTube Posts, and comments demonstrates that these groups usually employed ten different argumentation schemes, which are listed in Table 1. The most frequently used scheme was the *Argument from Sign*, where conclusions were drawn based on specific signs or pieces of evidence. The second most common scheme was the *Argument from Expert Opinion*, in which claims were supported by referencing experts within the relevant field. The *Argument from Consequences* ranked third, where the appropriateness of an action or policy was judged based on its outcomes. Also, in some cases, we found posts that did not follow any recognizable argumentation scheme, and these were categorized as *None*. We interpreted this to mean that these posts did not involve any form of logical reasoning.

**Table 2:** Argument schemes used in the posts about LK-99.

Category	Description	Examples
Expert Opinion	supports a claim based on the views of an expert.	“The verification committee of the Korean Society of Superconductivity and Cryogenics has concluded that it is not an ambient-pressure superconductor.”
Analogy	draws a conclusion based on the similarity between the two situations.	“Just as nuclear development has critical implications for neighboring countries, the discovery of LK-99 is argued to have similarly far-reaching impacts for societies that prioritize scientific and technological advancement.”
Cause to Effect	supports a claim using the relationship between cause and effect.	“It is positive in that it can maximize the transmission efficiency of power lines, reduce electricity costs, and decrease environmental pollution and carbon emissions by minimizing electrical losses.”
Sign	draws a conclusion based on certain signs or evidence.	“The entire world was thrown into upheaval. Not only in Korea, but related stocks in the U.S. and China surged, and platforms like YouTube and online communities are experiencing an unprecedented surge in interest in science.”
Example	draws a general conclusion through specific examples.	“It is based on the historical example of Einstein’s theory of relativity, which, despite lacking experimental evidence at the time of its announcement, was gradually accepted over time.”
Popular Opinion	supports a claim based on what many people believe.	“Superconductor technology has limitless applications, not only in semiconductors but also in electric vehicle batteries and more. There are optimistic projections that, if Korea were to develop a room-temperature superconductor, the world’s wealth could gravitate toward Korea.”
Consequences	judges the rightness or wrongness of an action or policy based on its consequences.	“This is because, if commercialized, it is a discovery expected to bring ground-breaking advancements to existing industries.”
Commitment	justifies current actions based on previous promises or statements.	“The Ministry of Science and ICT formed a review committee consisting of seven external experts from six fields: broadcasting, law, management, accounting, technology, and audience.”
Ignorance	claims something is true on the grounds that it has not been proven false.	“Because no decisive evidence has been presented to disprove LK-99’s potential as a room-temperature superconductor, some assert that it must, by default, be a valid breakthrough.”
Values	supports a claim based on specific values or ethical principles.	“It is recognized as a discovery significant enough to create a quantum leap in the scientific and industrial sectors, drawing a great deal of research and attention.”

**Fig. 4:**
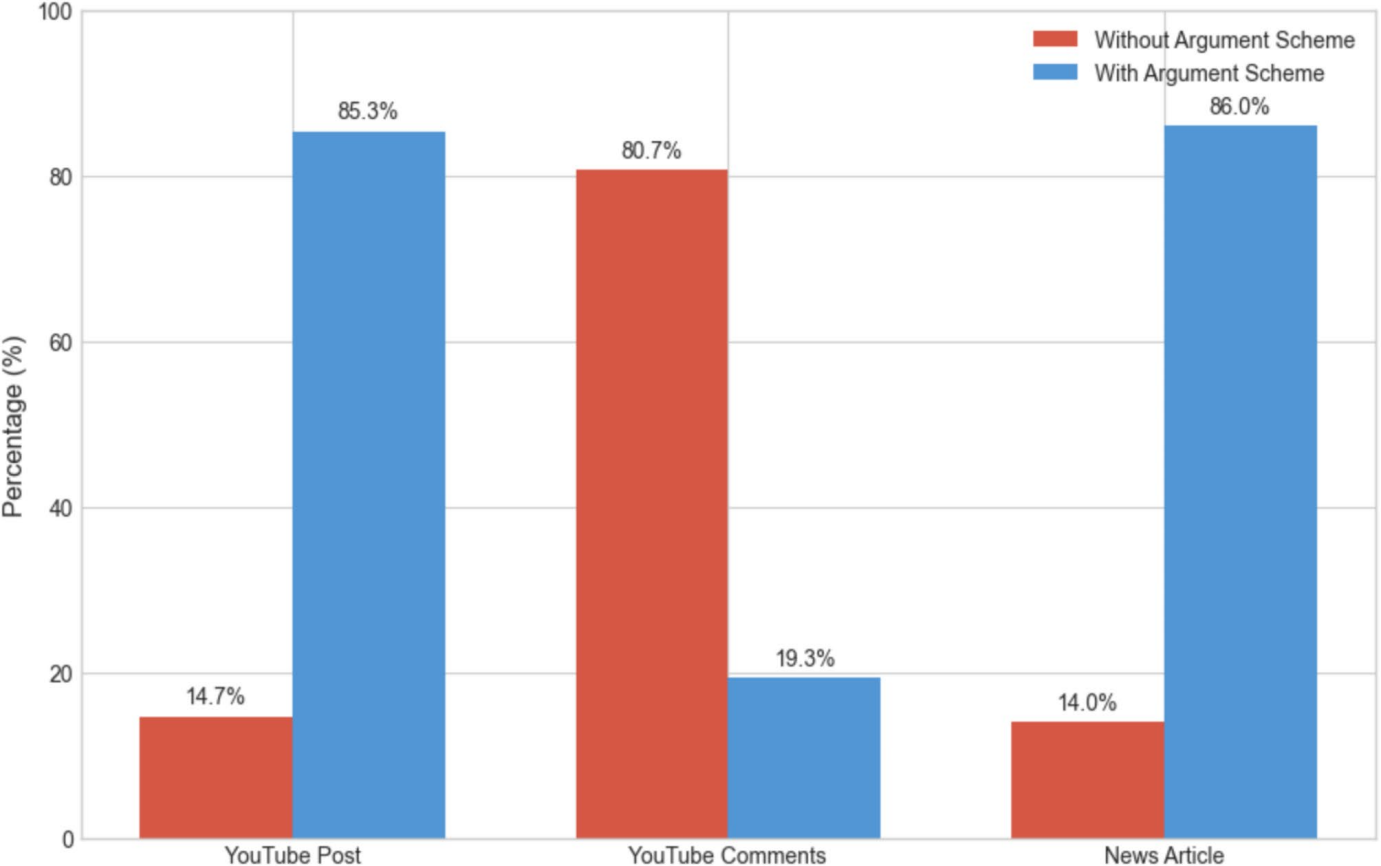
Comparisons of argumentation schemes among News articles, YouTube videos, and YouTube comments.

Figure 4 provides an overview of whether or not identifiable argumentation schemes are present in news articles, YouTube Posts, and comments. As shown, both news articles and YouTube Posts predominantly rely on argumentation schemes—especially *Argument from Sign*, *Argument from Expert Opinion*, and *Argument from Consequences*—while YouTube Posts also exhibit a slightly higher use of *Argument from Cause to Effect*. In contrast, 80% of YouTube comments lack any identifiable argumentation scheme, with the remaining 20% occasionally employing *Argument from Expert Opinion*,* Argument from Sign*, and *Argument from Consequences*. Although we observed minimal use of *Argument from Analogy*,* Argument from Commitment*, and *Argument from Values* across all three groups, these schemes are not individually depicted in Fig. 4 due to their low frequency. If further granularity is needed—such as breakdowns of specific argumentation schemes within each platform—a more detailed figure or supplementary table may be necessary.

**Fig. 5:**
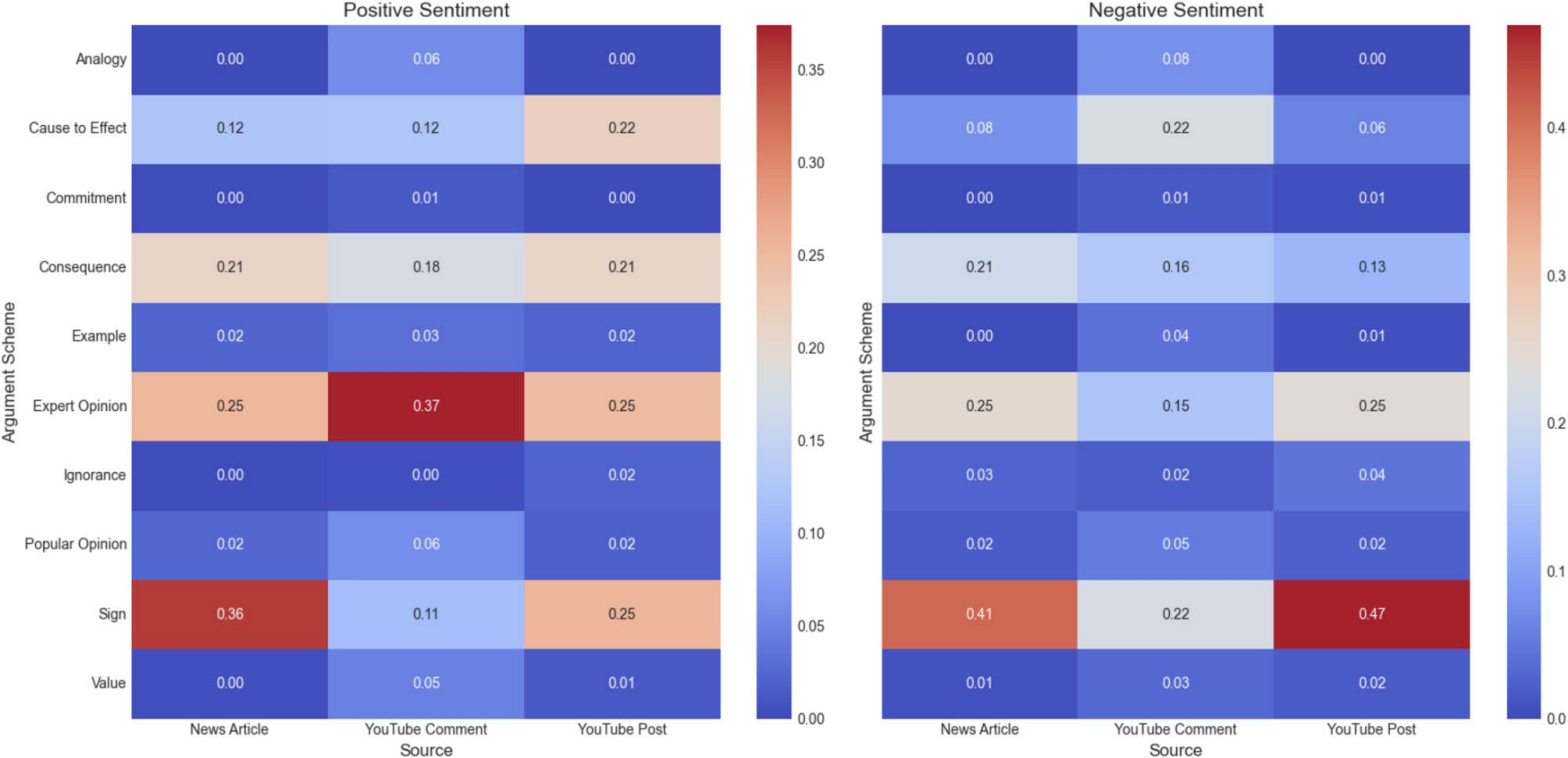
Association between sentiments and argumentation schemes.

To further explore how sentiments are linked to argumentation in the posts, we analyzed argumentation schemes based on positive, neutral, and negative sentiments. Figure 5 illustrates how different argumentation schemes were used in relation to positive and negative sentiments across news articles, YouTube posts, and comments. In our analysis, we conducted correlation tests to examine the relationships between sentiment polarity and the frequency of specific argumentation schemes. Our results indicate that several of these correlations were statistically significant (*p* < 0.05), underscoring the association between emotional tone and the use of particular reasoning patterns. In this figure, the closer the hue is red, the stronger the correlation. For positive sentiments, news articles frequently employed *Argument from Sign* and *Expert Opinion*, while YouTube Posts showed higher usage of *Consequences* and *Cause to Effect*. In YouTube comments, *Expert Opinion* was the most used scheme. For negative sentiments, *Argument from Sign*,* Expert Opinion* and *Consequences* dominated across all three groups, with YouTube posts showing the highest proportions for these schemes. YouTube comments also included *Cause to Effect* arguments more frequently in negative contexts.

**Fig. 6:**
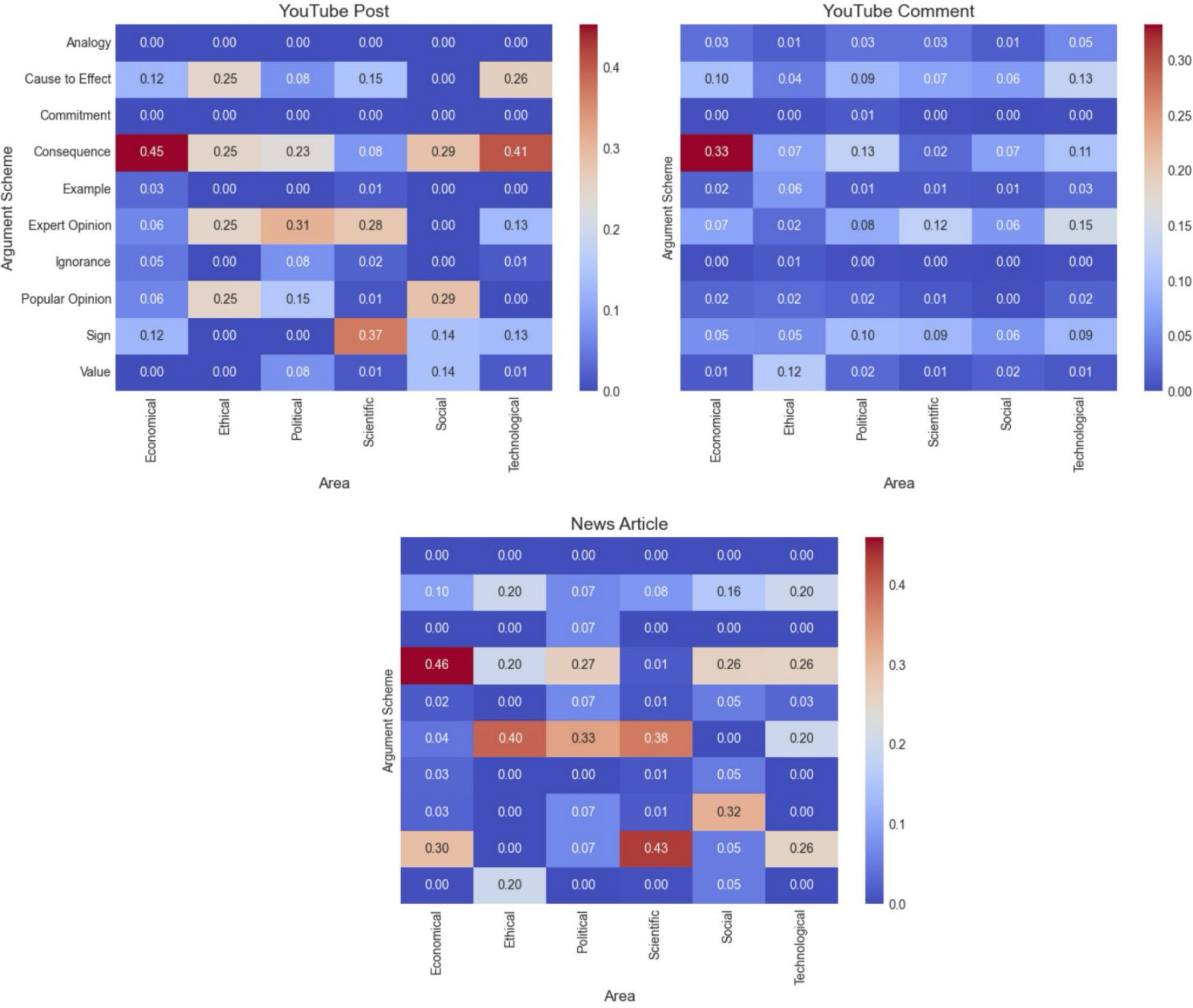
Heatmaps illustrating the frequency of each argumentation scheme across different sections for YouTube posts (top-left), YouTube comments (top-right), and news articles (bottom).

Additionally, we examined how the argumentation schemes used in news articles and YouTube Posts vary based on the sections where they are published. YouTube and online news platforms offer different sections, allowing content creators to select the most appropriate one to attract and interact with viewers who are likely to be more interested in the content. Figure 6 compares the distribution of argumentation schemes across different content sections for news articles, YouTube posts, and comments. The rows represent the ten argumentation schemes, and the columns indicate content sections (e.g., Economy, Political, Society, Science, Technology). The color scale corresponds to the relative frequency of each scheme within a given section, with red hues denoting higher usage and blue hues indicating lower usage. In news articles, scientific and economic sections prominently used *Argument from Sign* and *Expert Opinion*. The economic section also highlighted *Consequences* as a key argumentation scheme. YouTube posts in scientific sections mirrored news articles, frequently employing *Sign* and *Expert Opinion*. However, in technological and economic sections, they leaned toward *Consequences* and *Cause to Effect*. YouTube comments showed limited differentiation by section, though *Consequences* appeared more frequently in economic discussions.

## Discussion

The analysis of public discussions about LK-99 highlights complex dynamics that extend beyond simple media-public relationships. News articles demonstrated the most volatile sentiment trends, while YouTube comments maintained a stable, slightly negative orientation. This distinction suggests that public opinions on scientific issues are shaped by various factors beyond direct media exposure, as supported by recent studies^2^.

Notably, public comments often diverged in sentiment from media coverage. While news articles and YouTube videos emphasized the technological and economic potential of LK-99, comments consistently reflected skepticism, indicating a more evaluative and critical engagement with scientific claims.

The relative stability of public sentiment in comments, despite fluctuating media narratives, supports findings from climate change research, suggesting a growing resilience in public opinion formation^17^. This resilience may stem from increased scientific literacy or persistent skepticism toward unverified claims.

In our analysis, we observed that approximately 80% of YouTube comments lacked identifiable argumentation schemes, whereas only 14–15% of news articles and YouTube posts showed no discernible reasoning structure. We acknowledge that YouTube channels can be operated by both experts and non-experts. However, the higher incidence of comments without identifiable argumentation schemes suggests that, in practice, user-driven discourse on YouTube may often exhibit less structured reasoning than the more curated or editorially guided content found in news articles or certain YouTube posts. These findings highlight potential differences in how scientific information is processed across various media contexts, rather than strictly delineating professionals from non-professionals^1^.

However, the absence of formal argumentation schemes in comments does not necessarily indicate irrational engagement. Instead, it may reflect different modes of processing scientific information, as suggested by recent work on public understanding of science^9^. Publics’ more intuitive reasoning might be better suited for rapidly evaluating complex scientific claims in social media contexts.

The variation in argumentation schemes across different sentiment categories suggests that there may be some association between authors’ reasoning patterns and their emotional responses, although the observed correlations were generally weak to moderate. The prevalence of expert opinion and sign-based arguments in positive sentiment, as opposed to consequence-based reasoning in negative sentiment, indicates that how people argue about scientific issues is intrinsically linked to their emotional orientation toward those issues^4^.

The examination of science channels’ influence on YouTube raises important questions about the evolving landscape of science communication. Our channel-level analysis (see Table 1) reveals that among the top 20 channels by engagement, only three specialize in science and only two are traditional legacy media, while the majority of channels lack a direct scientific focus. In fact, although these non-specialist channels represent only 12.58% of the total LK-99-related videos, they attract 75.95% of all views and 77.98% of all likes. This pattern suggests a dominance of non-expert voices in LK-99 discussions, which reflects broader trends in science communication on social media^18^. These findings prompt further inquiry into how scientific understanding and public opinions are shaped by established scientific organizations versus the more heterogeneous voices on digital platforms. These findings suggest that within the platforms examined—news and YouTube—there is a notable divergence between traditional science communication channels and those channels that attract the majority of public engagement.

Furthermore, our research extends previous work on social media’s role in science communication by demonstrating how different stakeholders engage with scientific controversies^14^. The disconnect between media contents and public responses suggests that traditional approaches to science communication may need revision. While news outlets and YouTube creators often focused on the potential technological and economic impacts of LK-99, public comments showed more interest in verification and methodological concerns.

Based on our analysis and supported by previous research, our findings indicate that structured argumentation is often lacking in user comments, which may contribute to persistent public skepticism. Therefore, science communicators should engage directly with skeptical viewpoints to help bridge the gap between scientific evidence and public opinions—a strategy supported by studies demonstrating that acknowledging skepticism can improve trust and understanding^37,38^. In addition, our observation of variation in argumentation schemes suggests that no single messaging approach is universally effective. Employing multiple forms of argumentation—such as combining evidence-based expert opinions with relatable analogies and clear causal explanations—can better resonate with diverse audience segments and help mitigate polarization^9,10^. Our results also indicate that limited scientific literacy may contribute to the prevalence of unstructured discourse on social media. Enhancing critical thinking and improving public education on scientific topics can empower audiences to evaluate information more rigorously, thereby reducing the influence of misinformation^2,39^. Finally, our channel-level analysis shows that non-expert channels dominate public engagement, suggesting that traditional science communication channels might benefit from collaboration with influential digital platforms or popular content creators. Integrating the credibility of established experts with the wide reach of non-expert influencers could ensure that accurate scientific information reaches a broader audience^5,6^. Overall, these recommendations are grounded in our empirical results and corroborated by prior studies, highlighting the need for multifaceted communication strategies in the digital age.

Our study has several limitations that future research could address. First, our analysis focused only on the LK-99 controversy. More research would be needed to confirm the consistency of tpublics’ and media sources’ communication patterns, including multiple scientific controversies. Second, our analysis of argumentation schemes might not capture all forms of public reasoning, particularly those that do not fit traditional argumentation frameworks. A more in-depth analysis of publics’ reasoning strategies can be meaningful in understanding how they construct opinions based on the scientific information offered. Finally, our analysis of YouTube Posts and comments could be influenced by the algorithmic content recommendation function of the social media platform. The role of algorithmic content recommendation in shaping exposure to scientific information deserves further investigation.

This research contributes to our understanding of how publics form opinions around scientific controversies in the digital age, highlighting the complex interplay between media coverage, argumentation patterns, and public sentiment. Future research could explore how these dynamics vary across different scientific controversies and cultural contexts and how science communication strategies might be adapted to better serve public understanding in an evolving media landscape.

## Conclusion

This study analyzed public perceptions and argumentation patterns during the LK-99 superconductivity controversy across news articles, YouTube videos, and comments, revealing key trends in digital-age science communication.

Our analysis reveals several important insights. The overall sentiment observed in our dataset exhibits a degree of stability and appears to operate somewhat independently of media narratives, suggesting that diverse publics may adopt a critical and evaluative stance toward scientific claims. Additionally, our findings show variations in argumentation patterns across different media, with structured reasoning being less frequently identifiable in informal online discussions. Moreover, our channel-level analysis indicates that non-expert voices—as measured by engagement metrics on YouTube—tend to capture substantial audience attention compared to traditional science communication channels. We note, however, that these insights are derived from data collected from news articles and YouTube posts and comments, and further research is needed to more precisely understand the influence of specific actor groups on public discourse.

These findings underscore the need for adaptive science communication strategies that address public skepticism while balancing methodological transparency and engagement. Although our study did not directly assess public trust or scientific literacy, previous research indicates that enhancing public understanding is crucial for effective science communication. Therefore, future studies should explore strategies that not only highlight potential benefits but also actively work to improve public comprehension of scientific information.

Our research also highlights the changing landscape of science communication. The dominance of non-expert channels in scientific discussions on social media platforms, including YouTube, represents both challenges and opportunities. While this trend may undermine traditional scientific authority, it also presents opportunities to diversify science communication methods and reach wider audiences through more accessible and engaging formats.

In summary, our findings suggest that effective science communication should (i) directly address public skepticism, (ii) integrate multiple forms of argumentation to resonate with diverse audiences, (iii) enhance scientific literacy and critical thinking, and (iv) foster collaborations between traditional science communicators and popular digital influencers. These recommendations, which are grounded in our empirical results and supported by prior research, underscore the need for multifaceted communication strategies in the digital age.

Future research could explore how these patterns manifest across different scientific controversies, cultural contexts, and emerging media platforms. Additionally, investigating the role of algorithmic content recommendation in shaping exposure to scientific information could provide valuable insights for science communication strategies.

These findings contribute to our understanding of how scientific information is communicated and received in the digital age, offering practical implications for improving public engagement with science. As the media landscape continues to evolve, the ability to effectively communicate scientific information while maintaining credibility and engaging diverse audiences will become increasingly crucial for the scientific community.

## Data Availability

The datasets used and/or analyzed during the current study were collected from publicly available sources, including YouTube and the Korea Press Foundation’s BigKinds platform (bigkinds.or.kr). Data from YouTube were collected using publicly accessible APIs provided by Google. Additionally, the processed datasets generated and/or analyzed in this study are available from the corresponding author, Hunkoog Jho, upon reasonable request via e-mail at hjho80@dankook.ac.kr.
